# A Clinical Pilot Study of Individual and Group Treatment for Adolescents with Chronic Pain and Their Parents: Effects of Acceptance and Commitment Therapy on Functioning

**DOI:** 10.3390/children3040030

**Published:** 2016-11-16

**Authors:** Marie Kanstrup, Rikard K. Wicksell, Mike Kemani, Camilla Wiwe Lipsker, Mats Lekander, Linda Holmström

**Affiliations:** 1 Functional Area Medical Psychology, Functional Unit Behavioral Medicine, Karolinska University Hospital Solna, P8:01, 171 76 Stockholm, Sweden; Rikard.Wicksell@karolinska.se (R.K.W.); Mike.Kemani@karolinska.se (M.K.); Camilla.Wiwe-Lipsker@karolinska.se (C.W.L.); Linda.Holmstrom@ki.se (L.H.); 2 Department of Clinical Neuroscience, Karolinska Institutet, Nobels väg 9, 171 77 Stockholm, Sweden; Mats.Lekander@ki.se; 3 Stress Research Institute, Stockholm University, 106 91 Stockholm, Sweden; 4 Department of Women’s and Children’s health, Karolinska Institutet, H2:00, Karolinska University Hospital Solna, 171 76 Stockholm, Sweden

**Keywords:** cognitive behavior therapy (CBT), acceptance and commitment therapy (ACT), treatment, intervention, pain disability, persistent pain, adolescent

## Abstract

Pediatric chronic pain is common and can result in substantial long-term disability. Previous studies on acceptance and commitment therapy (ACT) have shown promising results in improving functioning in affected children, but more research is still urgently needed. In the current clinical pilot study, we evaluated an ACT-based interdisciplinary outpatient intervention (14 sessions), including a parent support program (four sessions). Adolescents were referred to the clinic if they experienced disabling chronic pain. They were then randomized, along with their parents, to receive group (*n* = 12) or individual (*n* = 18) treatment. Adolescent pain interference, pain reactivity, depression, functional disability, pain intensity and psychological flexibility, along with parent anxiety, depression, pain reactivity and psychological flexibility were assessed using self-reported questionnaires. There were no significant differences in outcomes between individual and group treatment. Analyses illustrated significant (*p* < 0.01) improvements (medium to large effects) in pain interference, depression, pain reactivity and psychological flexibility post-treatment. Additionally, analyses showed significant (*p* < 0.01) improvements (large effects) in parent pain reactivity and psychological flexibility post-treatment. On all significant outcomes, clinically-significant changes were observed for 21%–63% of the adolescents across the different outcome measures and in 54%–76% of the parents. These results support previous findings and thus warrant the need for larger, randomized clinical trials evaluating the relative utility of individual and group treatment and the effects of parental interventions.

## 1. Introduction

Chronic pain is common among children and adolescents and is related to disability for up to 80% of those affected, for example negatively affecting leisure or school activities [1,2]. Pediatric chronic pain is related to poorer family functioning and may lead to an economic burden for parents and society [3,4]. Hence, there is a great need for interventions that effectively improve patient and family functioning. Although current medical treatment has often proven to be insufficient in reducing symptoms and improving functioning in pediatric chronic pain patient, systematic review findings support the use of psychological treatments, particularly those based on cognitive behavior therapy (CBT) [5]. CBT commonly includes a broad spectrum of interventions, based on different theoretical perspectives and models, aimed at both reductions in pain and distress, as well as improvement in overall functioning. Although there is evidence for the efficacy of CBT to treat pediatric chronic pain, debilitating symptoms can still remain for a number of patients, and therefore, there is a need to better understand change processes. Furthermore, considering the need for further research of the effects on functioning and mood [5], interventions aimed at decreasing pain interference (i.e., the impact of pain on functioning) and emotional reactivity to the pain experience, are crucial. Acceptance and commitment therapy (ACT) constitutes such a development within CBT [6]. From an ACT perspective, a primary source of disability and reduced quality of life is the inflexible use of avoidance strategies (psychological inflexibility), especially in the presence of pain and distress. Therefore, the key treatment target in ACT is to increase psychological flexibility (i.e., the ability to actively choose and act in line with long-term goals and values, even when in the presence of disturbing symptoms), by undermining the dominance of distressing thoughts, emotions and physical sensations, using various techniques, such as defusion and acceptance [6]. This specific focus on increasing behaviors in the direction of functionality in important life areas despite ongoing pain is consistent with the Pediatric Initiative on Methods, Measurement, and Pain Assessment in Clinical Trials (PedIMMPACT) recommendations [7], which emphasize the importance of targeting pain-related functioning in physical, emotional and social domains for treatments of pediatric chronic pain. Today, the empirical support for ACT for adult, unspecific chronic pain is strong [8]. However, only a few studies so far have been conducted analyzing children and adolescents.

Of these studies, findings from three ACT-based outpatient interventions for pediatric chronic pain by Wicksell et al., include an RCT consistently illustrate improvements in functioning [9,10,11]. In the RCT it was shown that these improvements in functioning at follow-up were mediated by improvements in pain impairment beliefs and pain reactivity, which are two variables strongly associated with psychological flexibility [12]. Similarly, results from a study evaluating an ACT-based intensive inpatient-program [13] showed that adolescents reported improved functioning along with increased levels of acceptance. A recent study by Ghomian and Shairi [14] on ACT for young children with chronic pain found increased functional ability after treatment compared with the control group. Furthermore, a pilot study on pediatric patients suffering from chronic pain and neurofibromatosis type 1 [15] showed that ACT may decrease pain interference.

Notably, previous interventions for adolescents have been delivered both individually [9,10,11] and in a group format [13,15], but to date, there is no scientific evaluation comparing the relative utility of these treatment formats. Furthermore, despite the evident relationship between pediatric chronic pain and family functioning, studies evaluating the effects of parental support programs on parent behaviors and distress are scarce [16]. Recent studies suggest the importance of ACT-consistent behaviors in parental management of pediatric chronic pain. For example, parental psychological flexibility has been found to correlate positively with adolescent acceptance and negatively with an adolescent pain-related impact on functioning and dysfunctional parental responses [17]. Therefore, parent psychological flexibility is suggested to be a potentially important target for the treatment of children and adolescents with chronic, debilitating pain [17,18]. In addition, considering the impact of pain on family functioning [3], parent emotional reactivity to child pain should also be explored. To date, one study has explored the utility of ACT for parents of children suffering from chronic pain [15], and the results suggest that interventions may improve parent acceptance.

Thus, there is preliminary support for the use of ACT to treat pediatric chronic pain, but more research is needed to further investigate the effects of ACT interventions in children and adolescents with chronic pain conditions. Furthermore, more studies are needed to evaluate the effects of ACT-based parental support programs. Positive effects have been seen in both individually- and group-delivered ACT interventions although these formats have not been compared using the same protocol. This is an important clinical question, as services might be providing both formats. Hence, the aims of the present clinical pilot study were to preliminarily evaluate the effects of (1) an ACT-based intervention provided in a group or in an individual format to adolescents with disabling chronic pain conditions, on functioning (i.e., pain interference, pain reactivity, depressive symptoms and functional disability), pain intensity and psychological flexibility; and (2) an ACT-based parental support program on parent emotional functioning, pain reactivity and psychological flexibility.

## 2. Methods

### 2.1. Study Setting and Design

This pilot study was performed by a team of five psychologists, two pain physicians and one physiotherapist, at a tertiary pain specialist clinic in Stockholm, Sweden. Patients between 12 and 18 years referred to the clinic due to chronic debilitating pain and their parents were considered eligible for inclusion in this study. Participants were randomized to either group (*n* = 24) or individual (*n* = 24) ACT treatment. The randomization sequences were generated via an online randomization service accessible at https://www.random.org/. An administrator who was not involved in treatment delivery randomized the participants, placed the information in coded sealed envelopes and informed the participants about which condition they had been assigned to by opening the envelopes in their presence. Data collection began in 2009 and ended in 2012. Pre-treatment assessments from study participants were included as part of previously-reported cross-sectional studies [19,20]. This clinical pilot trial was not registered.

### 2.2. Participants

Inclusion and exclusion criteria were assessed in semi-structured clinical interviews during the first visit to the clinic. Patients were included if they (1) were referred to our tertiary care pain clinic; (2) were between 14 and 18 years old (initial criterion 12 and 18 years, see below); (3) had suffered from pain >6 months; (4) reported insufficient effects of previous pain treatments; (5) reported substantial pain-related disability. They were excluded if (1) improvement was expected without treatment (e.g., had improved between referral and assessment); (2) psychiatric co-morbidity was considered the main reason for disability, required immediate intervention or was assumed to interfere with the planned intervention; (3) there was a substantial risk for suicide; (4) they had substantial cognitive dysfunction or reduced proficiency in Swedish; (5) they had other on-going or planned treatments (i.e., within the next 6 months); (6) pain was recurrent rather than continuous (defined as ≥4 completely pain-free days per week); and (7) pain was fully explained by a pathophysiological process, e.g., cancer. Adolescents not eligible for the study, who declined participation (e.g., those who did not want to take part in randomization) or who dropped out (e.g., those who changed their mind after being randomized to group treatment), were offered standard treatment at the clinic.

Parents of included adolescents were invited to participate in the parent support program, and all included adolescents had at least one parent who participated in the study. Parents data were collected from the same parent at all time points, although both parents, if applicable, were participating in the parent sessions.

**Figure 1:**
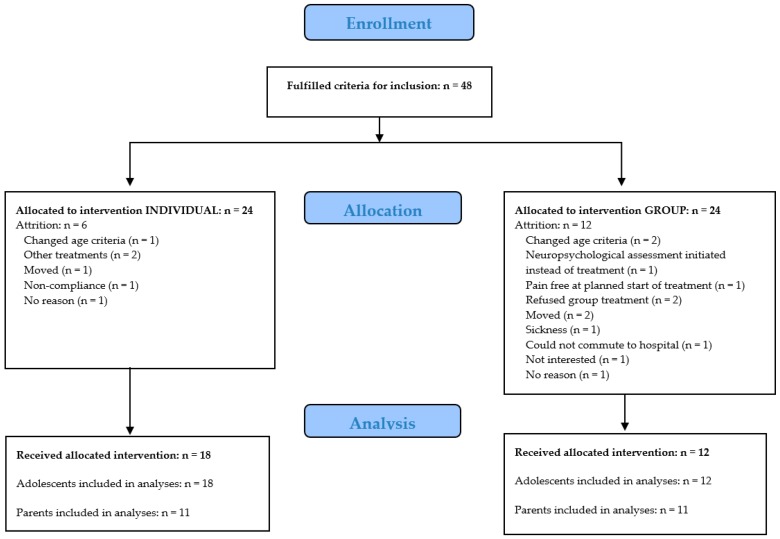
Flow diagram. Changed age criteria: researchers initially aimed to include participants from the age of 12 years, but due to low inflow of younger adolescents, the age range was set to 14–18 years, and three randomized participants were excluded.

As shown in Figure 1, 48 adolescent-parent dyads were randomized (24 participants randomized to individual treatment and 24 participants to group treatment). Attrition rates were 6 and 12 participants for the individual treatment condition and group treatment condition, respectively. Reasons for attrition are reported in the flow chart (Figure 1). The group condition included four groups, with 2–4 participants per group at post-treatment. At post-treatment assessment, 30 adolescents remained in the study, and these were included in the analyses. Out of all included parents, 28 parents provided data. Six parents were excluded from analysis because ratings were performed by different parents at different time-points (i.e., mother ratings at pre- and mid-, but father ratings at post-assessment). Therefore, data of 22 parents were included in the analyses.

### 2.3. Intervention

The intervention consisted of an interdisciplinary ACT-based outpatient treatment based on a protocol from a previous RCT with 10 adolescent sessions and one to two parent sessions [10]. In the current protocol, we increased the involvement of the pain physician, included two sessions with a physiotherapist to address pain interference in regard to physical activity, and added sessions conducted by the psychologists for both adolescents and parents. This resulted in a total of 18 sessions (Table 1). The majority of sessions were conducted by a psychologist.

The treatment can be divided into four phases with different, although related, treatment objectives: (1) preparing for behavior change (including pain education, conducted separately with parents and adolescents by a pain physician); (2) shifting perspective (i.e., changing the focus from pain reduction to a valued life); (3) acceptance (i.e., willingness to experience symptoms and thoughts without trying to change them) and cognitive defusion (i.e., have a perspective on and step back from thoughts and feelings); and (4) values-oriented behavior activation (i.e., behavior change in the service of living a meaningful life). All phases included the use of age-appropriate metaphors and experiential exercises and an emphasis on functional restoration and behavior activation. Importantly, although two sessions focused specifically on the processes of acceptance and defusion, these behavioral strategies were addressed and practiced continuously during all phases of the treatment. In every session, participants were given individualized home assignments related to the treatment content and to their own specific challenges, and these outcomes were discussed in the beginning of the following session.

The parent support program was embedded in the treatment and comprised of four sessions (Sessions 3, 6, 11 and 12). Session 12 was a joint session, with both adolescents and parents participating in continued pain education and discussion about pain and symptoms, in relation to values-oriented behavioral activation. In short, the objective of the parent support program was to improve the parents’ ability to use values- and acceptance-based coaching behaviors, in order to support their child to increase functioning, even in the presence of pain and distress. Parents were given home assignments between Parent Sessions 6 and 11.

The intervention was carried out either individually or in a small-group format, with the same treatment content in both conditions. Group sessions were 2 h including a break, and individual sessions were 45 min. Group sessions were longer, in order to allow participation from all group members. Both adolescent and parent sessions were conducted in a parallel format (i.e., parents in the group treatment arm received group-based parent support sessions, and parents in the individual treatment arm received individual parent support sessions). If a participant in either condition missed a session, a short summary was individually given before the start of the next session.

Treatment fidelity was assured via the use of the detailed protocol, and all five psychologists involved in delivering treatment had formal training in CBT and ACT, with varying years of experience working with pediatric chronic pain patients. All psychologists were continuously supervised by a senior researcher with extensive experience using ACT for pediatric chronic pain.

### 2.4. Assessment

Data collection took place at pre-, mid- and post-assessment. Demographic and medical information (e.g., pain duration, pain location, temporal aspects of pain and pain medication) was collected in semi-structured screening interviews. Parents provided background information regarding educational status and work, through a self-reported questionnaire. Adolescent and parent functioning was assessed through self-reported questionnaires at all time points.

#### 2.4.1. Adolescent Measures

##### 2.4.1.1. Pain Intensity

Current pain intensity for all time points was rated on a numerical rating scale from 0 (no pain at all) to 6 (extreme pain).

##### 2.4.1.2. Pain Interference Index (PII)

The Pain Interference Index (PII) was used to assess the influence of pain on behaviors, or to what extent pain impacts everyday functioning. PII consists of six items, concerning whether pain has made it difficult to do schoolwork, leisure activities, spend time with friends, has affected mood, physical activities or sleep. Items are rated on a scale from 0 (not at all) to 6 (completely), and the maximum score is 36. The Swedish version of PII has shown sensitivity to change [10], along with satisfying reliability and internal consistency [20]. It has also been used in an English version for parents, providing further support for the consistency and validity of the instrument [21]. At pre-treatment assessment, Cronbach’s alpha for PII in this sample was 0.82.

##### 2.4.1.3. Pain Reactivity Scale (PRS)

The Pain Reactivity Scale (PRS) measures worry and general emotional reactivity to pain [10]. It contains five items: (1) How often do you worry about your pain? (2) How often are you worried that you will not be able to do things because of your pain? (3) How often are you worried that you will not be able to do things in the future because of your pain? (4) How difficult do you think it is to think about things that are related to your pain? (5) How often are you angry or said because it hurts? PRS is rated on a scale from 0 (never/not at all) to 6 (always/very much), with a maximum total score of 30. The instrument has shown sensitivity to change in ACT-treatment [10]. At pre-treatment assessment, Cronbach’s alpha for PRS in this sample was 0.83.

##### 2.4.1.4. Center for Epidemiological Studies Depression Scale Children (CES-DC)

The Center for Epidemiological Studies Depression Scale Children (CES-DC) [22,23] was used to assess depressive symptoms. The scale consists of 20 items concerning feelings and actions relevant for depressive disorder, and they are rated on a scale from 0 (not at all) to 4 (a lot), with four reversed items. The maximum score is 60. CES-DC has shown adequate psychometric properties and the ability to discriminate depressive disorder in a Swedish population of adolescents, where a cut-off of 24 was proposed [24]. At pre-treatment assessment, Cronbach’s alpha for CES-DC in this sample was 0.90.

##### 2.4.1.5. Functional Disability Index (FDI)

The Functional Disability Index (FDI) [25] was used to assess child functioning. FDI has been evaluated for pediatric patients with chronic pain, with evidence of reliability and validity [26], and it is recommended by the PedIMMPACT as a core outcome measure of physical functioning [7]. The instrument has 15 items rated on a scale from 0 (no problems) to 4 (impossible), and the maximum score is 60. There are established cut-off scores for the FDI, with 0–12 indicative of no/minimal disability, 13–20 indicative of mild disability, 21–29 indicative of moderate disability and scores of 30 or over indicative of severe disability [27]. In the present study, we used the parent version, which has been found to correlate significantly with child ratings [26]. At pre-treatment assessment, Cronbach’s alpha for FDI-P in this sample was 0.93.

##### 2.4.1.6. Psychological Inflexibility in Pain Scale (PIPS)

The Psychological Inflexibility in Pain Scale (PIPS) is a measure of psychological inflexibility (i.e., the inability to carry out behaviors in line with a valued life when pain, unpleasant thoughts or emotions are present) [6]. PIPS consists of 12 items rated on a scale from 1 (never true) to 7 (always true), and the maximum score is 84. Examples of items are: “I would do almost anything to get rid of my pain” and “it’s not me that controls my life, it’s my pain”. Previous research with adults with chronic pain supports the psychometric properties and the use of PIPS as a process measure to assess changes in pain-related disability after ACT-based treatment [28,29,30]. At pre-treatment assessment, Cronbach’s alpha for PIPS in this sample was 0.85.

#### 2.4.2. Parental Measures

##### 2.4.2.1. Hospital Anxiety and Depression Scale (HADS)

The Hospital Anxiety and Depression Scale (HADS) [31] was used to measure parental levels of anxiety and depressive symptoms. The scale consists of 14 items divided into two subscales, HADS-A and HADS-D, with seven items respectively. Agreement is rated on a scale from 0 to 3, for example “I still enjoy the things I used to enjoy” most of the time (0), a lot of the time (1), from time to time, occasionally (2) or not at all (3). A score between 0 and 7 on each subscale respectively indicates non-cases of anxiety or depressive disorder; a score between 8 and 10 indicates possible cases; and scores of 11 and above indicate probable cases (i.e., a score >8 on each scale respectively defines caseness) [32]. HADS has been used extensively across both patient and general populations with results in favor of using the instrument for both identification of caseness and of symptom severity [32], and results from a study in the general population support the validity of the Swedish version used in this sample [33] At pre-treatment assessment, Cronbach’s alpha for HADS in this sample was 0.82.

##### 2.4.2.2. Pain Reactivity Scale Parent (PRS-P)

The Pain Reactivity Scale described above was also used for parents (PRS-P) to measure their emotional reactivity to their child’s pain. Parents were asked the same questions as in PRS, but in relation to their child, e.g., “How often do you worry about your child’s pain”. At pre-treatment assessment, Cronbach’s alpha for PRS-P in this sample was 0.91.

##### 2.4.2.3. Parent Psychological Flexibility Questionnaire (PPFQ)

The Parent Psychological Flexibility Questionnaire (PPFQ) was developed with the aim of measuring parental psychological flexibility in the context of pediatric chronic pain [17]. Further development of the instrument supported the validity and reliability of a shorter, 17-item version, and the authors suggest its potential for assessment of parent psychological flexibility in treatment interventions for pediatric patients with chronic pain [18]. Items are rated on a scale from 0 (never true) to 6 (always true), and examples of items are: “Even though my child has pain we can continue to do things that are important and enjoyable” and “My child’s pain makes it impossible to focus on anything else” (reversed scoring). In this study, we used a 10-item version of the instrument, with seven reversed items and a maximum score of 60, on the basis of a psychometric evaluation and factor analysis of a Swedish version of the instrument [34]. At pre-treatment assessment, Cronbach’s alpha for PPFQ in this sample was 0.82.

### 2.5. Data Management

Throughout the whole dataset, 12 (<1%) items were missing completely at random (MCAR). These were manually imputed via person mean imputation, in order to maximize *n* for all analyses.

### 2.6. Data Analysis

Due to the fact that the data was non-normally-distributed, a non-parametric approach was applied. For a comparison of non-completers versus completers, and for comparisons of conditions (group versus individual) across all assessments, Mann–Whitney U tests were used. To assess when changes occur (pre–mid-assessment, mid–post assessments and pre–post-assessment), Wilcoxon signed-rank tests were used. Effect sizes were calculated from *z*-scores and the number of total observations by use of *r* [35]. Correlational effect sizes above 0.10 indicate a small effect, above 0.30 a medium effect and above 0.50 a large effect, according to Cohen [36]. Mean-based statistics were used to calculate clinically-significant changes for all significant outcome variables and pain intensity using the 1991 Jacobson–Truax method for reliable change, where a change of two standard deviations (*SD*) in the direction of functionality from the mean of the population under investigation is considered clinically significant [37,38]. Pairwise deletion was chosen for all analyses, and all analyses were conducted with SPSS 23 and Excel 2011 for Mac. The significance level was set at a conservative level of *p* < 0.01 to account for multiple comparisons.

### 2.7. Ethical Considerations

All participants (parents and children) were given oral and written information about the study and provided signed informed consent. The study was approved by the Ethical Review Board in Stockholm, Sweden (2009:815:31/4, approved 2009/4:6).

## 3. Results

### 3.1. Initial Analyses

To assess potential differences between completers and non-completers/exclusions at pre-treatment assessment (*n* = 48), a series of Mann–Whitney U tests was performed. No statistically-significant differences in the pre-treatment assessments were found between children and parents who dropped out or were excluded, and children and parents who completed the study for any of the variables included in further analyses (PII, PRS, CES, FDI-P, PIPS, actual pain intensity and pain duration and parent HADS (total and subscales), PRS-P, PPFQ and parent chronic pain). All further analyses are based on the final sample of completers (*n* = 30).

### 3.2. Descriptive Statistics

The final sample included 30 adolescent participants (24 girls), with a mean age of 16 years and mean pain duration of more than four years. The majority of adolescents reported continuous pain from multiple sites. The parent sample included 28 parents (24 mothers), with a mean age of 47 years. Demographic and medical information is provided in Table 2.

### 3.3. Initial Analyses: Comparison of Group and Individual Treatment

For adolescents, no statistically-significant differences were found between the conditions at baseline regarding pain duration (*p* = 0.409) or prevalence of parent chronic pain (*p* = 0.209). Similarly, there were no significant differences between the conditions in any of the outcome variables at any of the time points (pain interference, pain reactivity, depression, functional disability, psychological inflexibility and pain intensity, *p =* 0.109–1.00). For parents, no statistically-significant differences were found between the conditions for any of the outcome variables at any of the time points (anxiety and depression and parent pain reactivity and psychological flexibility, *p* = 0.022–0.961). Table 3 shows median and min-max values for the total sample and group and individual conditions, respectively, for each time point. Further evaluations of treatment effects concern the total sample (*n* = 30) if not stated otherwise.

### 3.4. Effects of ACT-Treatment on Adolescent Functioning, Psychological Flexibility and Pain

Pain interference was significantly higher at pre- than at post-treatment assessment, median (Md) = 24.5 versus Md = 12.5, respectively, *p* < 0.001. The effect size was large (*r* = 0.51). Pain reactivity was significantly higher at pre- than at post-treatment assessment (Md = 21.5 versus Md = 13.0, respectively, *p* < 0.001), with a medium effect size (*r* = 0.49). Depressive symptoms were significantly higher at pre- than at post-treatment assessment (Md = 28.0 versus Md = 20.0, respectively *p* = 0.004), with a medium effect size (*r* = 0.37). FDI-P-scores did not change significantly from pre- to post-treatment assessment (Md = 15.5 versus Md = 6.0, respectively, *p* = 0.032), but a medium effect size was seen (*r* = 0.35). Psychological inflexibility was significantly higher at pre- than at post-treatment assessment (Md = 54.0 versus 37.0, respectively, *p* < 0.001), with a large effect size (*r* = 0.59). As expected, given the treatment objective in ACT, there were no significant changes in pain intensity at any of the time points (*p* > 0.608). Details on median and min–max values are provided in Table 3, and *z*-scores, *p*-values and effect sizes are listed in Table 4.

### 3.5. Effects of ACT-Treatment on Parent Anxiety, Depression, Pain Reactivity and Psychological Flexibility

No significant changes were seen in parent emotional functioning overall or anxiety and depression separately (*p* > 0.413). Improvement was seen in parent pain reactivity, with significantly higher ratings at pre- than at post-treatment assessment (Md = 22.5 versus Md = 15.0, respectively, *p* < 0.001), illustrating a large effect size (*r* = 0.57). Parent psychological flexibility was significantly increased from pre- to post-treatment assessment (Md = 49.5 versus Md = 67.0, respectively, *p* < 0.001) with a large effect size (*r* = 0.62).

### 3.6. Analyses of Temporal Change Patterns

Significant changes from pre- to mid-treatment assessments were only found for functional disability (pre–mid change *p* = 0.008). From mid- to post-assessments, however, the changes were significant in all other variables with significant overall treatment effects. Thus, the pattern of results illustrates more significant changes from mid- to post- than from pre- to mid-treatment assessments.

### 3.7. Clinically Significant Changes

#### 3.7.1. Adolescents

Table 4 shows the number of participants who have a clinically-significant change (≥2 *SD*) in the direction of functionality. A clinically-significant change in pain interference from pre- to post-treatment assessments was found for 47% of the participants, and a clinically-significant change in pain reactivity was found for 48%. Clinically-significant reductions of depressive symptoms were reported by 39%. For functional disability, improvement was seen in 21% of participants, and for pain intensity, 15% reported a clinically-significant reduction. Finally, 63% of participants reported a clinically-significant change in psychological flexibility. There were no significant differences between the conditions regarding the number of participants with clinically-significant improvement from pre- to post-treatment assessment for any of the variables (*p* between 0.083 and 1.00), but for depressive symptoms, there was a trend towards a significant difference (*z =* −2.41, *p* = 0.028), with more participants reporting clinically-significant reductions in depressive symptoms in the individual condition.

#### 3.7.2. Parents

Parent reactivity to child’s pain changed to a clinically-significant degree in the direction of functionality for 76% of the parents (Table 4). Similarly, 54% of parents reported clinically-significant increases in psychological flexibility. There were no significant differences between the conditions regarding the number of participants with clinically-significant improvement in neither parent pain reactivity nor psychological flexibility (*p* = 0.198).

### 3.8. Deterioration from Pre- to Post-Treatment Assessment

As seen in Table 4, a few adolescent participants reported deterioration (i.e., a change of ≥2 *SD* from pre- to post-treatment assessment). Deterioration in pain reactivity was reported by 7% of participants. In depressive symptoms, 7% also reported deterioration, and in functional disability, deterioration was seen in 11% of the adolescents. Deterioration in pain intensity was reported by 4% of participants. However, deterioration was not reported in parent pain reactivity or psychological flexibility.

## 4. Discussion

This clinical pilot study aimed to evaluate (1) the effects on functioning of an ACT-based intervention provided in a group or in an individual format to adolescents with disabling chronic pain conditions; and (2) the effects of a parental support program on parent functioning. Results showed improvements in adolescent functioning, as well as in parent psychological flexibility, with clinically-significant changes in the direction of a better functionality for a large portion of both adolescent and parent participants. Taken together, our findings of improved functioning in adolescents are consistent with previously-published studies on ACT for pediatric chronic pain conducted by our research group, as well as by researchers in other settings [9,10,11,12,13,14,15]. They are also in line with findings on improved functioning post-treatment in a systematic review on psychological therapies for pediatric chronic pain primarily based on CBT (including 37 studies, but only one ACT study) [5].

In addition to our aims, a preliminary comparison of treatment formats was conducted, which did not show any significant differences in effects between group and individually-delivered ACT treatment. However, as the sample is too small for an adequately-powered study with a non-inferiority design, the results should be seen as highly tentative, and larger studies are warranted in order to investigate the relative utility of these treatment formats. The pattern of changes between assessment points illustrated that changes occurred primarily from mid- to post-assessments. These results are interesting, as they suggest that treatment may be effective also when no or marginal improvements are seen in the first place. However, the design of the present study does not allow for any causal interpretation, and this pattern should merely be seen as indicative of the importance for future studies, including component analyses of treatment content, as well as the dose-response relationship.

In treatment of pediatric chronic pain, an overarching goal should be to decrease the impact of pain on a wide array of functioning outcomes [7,39]. The reported pre- to post-treatment assessment changes in pain interference scores and pain reactivity scores are in line with this goal. Similarly, the decreases in depressive symptoms from high pre-assessment scores in the current study (CES-DC Md = 28, which can be compared to the recommended cut-off of 24 for depressive disorder [24]) fits this pattern. Depression and functional disability commonly co-occur in chronic pain [40,41], and treatments that successfully target depressive symptoms are likely to have an important impact on overall pain-related functioning. Findings from recent systematic reviews on treatment for pediatric chronic pain [5,42] highlight the limited evidence for effects on depression in both psychological therapies overall and in intensive interdisciplinary treatment. Our results support previous findings from our research group regarding improvement in depressive symptoms (medium effect sizes) [10], suggesting that ACT treatment may hold promise in effectively targeting pain-related emotional dysfunction.

In this study, significant changes were only observed from pre- to mid-treatment assessment in functional disability (FDI-P), with baseline scores being relatively low as compared to PII-scores. This is consistent with previous findings [20] and suggests that FDI due to item content (e.g., “difficulties watching TV”) does not fully capture pain-specific concerns experienced by many patients. Adolescents in our study also reported significant changes in psychological flexibility, indicating that they were dealing with private events, such as pain and emotional distress in a more efficient way post-treatment. For example, instead of shutting the blinds and taking a rest, an adolescent with headache, fatigue and the thought “I can’t do it, I’d better wait until I feel better” (pain, distress and thoughts that dictate avoidance) observes these private events, decides not to act in accordance with short-term symptom relief and instead accepts symptom presence and chooses to pick up the phone and call a friend (exposure to previously-avoided situation and achievement of behavioral goal) because friendship is important (life value) and therefore worth pursuing.

This study provides an important contribution to the existing body of research on ACT-treatment for pediatric chronic pain, through the examination of clinically-significant changes in outcomes. In line with PedIMMPACT recommendations [7], pain intensity was included as a treatment outcome. While our finding for paint intensity, namely that changes in pain were not seen post-treatment, are in contrast to psychological therapies overall [5], they are still consistent with ACT-specific predictions [43]. Additionally, using the literature suggested cut-off of 2 *SD* in the direction of functionality [37], a large proportion of patients reported clinically-significant outcomes. It should be considered, however, that this cut-off is arbitrary and, in light of the severity of functional impairment and high chronicity of the population under investigation, may be seen as conservative. For example, a cut-off set at 1 *SD*, as suggested in adult chronic pain trials [44], would have rendered different results. What constitutes a clinically meaningful improvement in functioning for an adolescent with longstanding, debilitating pain should be further discussed and addressed in future trials.

The inclusion of parents in psychological therapies for pediatric chronic pain is encouraged, both on the basis of related family impact and dysfunction [3,4], and on the basis of existing evidence on child outcomes, but also because there is a need for more research concerning parent mental health and parent behavior outcomes [16,45]. That is, in addition to evaluating the importance of both overt parent behaviors and parent psychological states in relation to child dysfunction, such as observable dysfunctional parent behaviors [46] and self-reported parent behaviors and parent distress that are predictive of child distress and functioning over time [47], more studies are needed that specifically examine parent outcomes after treatment. One recent and important contribution is an Internet-delivered evaluation of family-based CBT for adolescents with chronic pain and their parents, where increased parent mental health and decreased dysfunctional parent behaviors were seen [48]. Similar to psychological therapies overall, there are only a few ACT studies evaluating parent support for parents of children and adolescents with physical health concerns [49], but findings indicate that ACT can be beneficial regarding parental adjustment for parents of children with autism [50] and can improve both child and parent outcomes in families where the child has traumatic brain injury [51] or cerebral palsy [52]. An adapted ACT-parent support program was carried out with parents of children with life-threatening illness, and parents reported decreased distress and increased psychological flexibility and mindfulness after participation [53]. Although parents have been involved in previous trials of ACT for pediatric chronic pain, to our knowledge, only one previous study exists specifically examining the effects of ACT support for parents of pediatric chronic pain patients [15]. Our study, evaluating the effects of a brief and structured ACT-based parent support program on parent mental health, parent pain reactivity and parent psychological flexibility, is therefore an important addition and extends previous findings. A majority of parents in our study reported clinically-significant improvements in dealing with their child’s pain post-treatment, indicating that both the novel variable parent pain reactivity and parent psychological flexibility, which have previously been reported on [17,18], in relation to child’s pain may be important treatment targets for enhancing effective parent behaviors. Regarding parent mental health, the parents in our study tended to be in the “possible cases” range of HADS at pre-treatment assessment (i.e., possibly suffering from anxiety) [32]. They reported no significant changes over the course of treatment. Future studies could include treatment content more specifically aimed at parent depressive or anxious symptoms, as such problems are commonly reported by parents of children with chronic pain [45,54] and potentially hamper effective parent behaviors for a subset of parents. Furthermore, chronic pain is common in parents of children with chronic pain [55], which is also seen in our sample, and the interference of parent chronic pain on parent behavior [56] and parent mental health should therefore be examined further.

A number of limitations should be considered when interpreting the results from this clinical pilot study. Due to the small sample, there was not enough power to adequately evaluate the relative effects from group versus individual treatment, and we suggest this as a focus for future studies. As in most clinical trials, improvements may partially be due to spontaneous recovery or other unspecific factors. However, spontaneous improvements in functioning for this sample with their long mean pain duration (>4 years) and level of disability would not be expected. Notably, our sample is similar regarding, e.g., pain duration as seen in other studies, which indicates representativeness and generalizability [42]. Furthermore, the adolescents in our study presented with relatively higher scores of depressive symptoms [57]. The representativeness of the sample was also seen in parents, as they appear similar to parent samples in other studies on pediatric chronic pain in tertiary care, regarding age and gender [13], as well as in anxiety and depression [54]. Problems with recruitment and retention in clinical trials have been described in the literature [58] and account also for this study where attrition was very high. In the present study, unexpected difficulties occurred after randomization to individual and group treatment, with two participants changing their mind after being allocated to the group condition and, thus, not initiating treatment. In addition, some parent assessments had to be excluded from the analyses due to different parents completing the questionnaires (i.e., when analyzing data, it was found that the mother conducted the rating at baseline and the father at post-assessment). Apart from following a structured protocol and regular supervision, no fidelity checks were performed in this clinical study, e.g., analyses of videotaped sessions. This is advisable for future studies to ensure protocol adherence and that sessions are performed in accordance with ACT theory. Furthermore, for adolescent pain interference and functional disability, the adolescent version of the PII and the parent version of the FDI were used when ideally both parent and adolescent ratings of both these outcomes should have been included. Finally, instead of measuring current pain intensity, a more expanded assessment, e.g., using a daily pain diary for a shorter period, would have given more detail and thereby also more confidence in regards to findings.

Compared to medical or surgical interventions aimed at pain relief, the potential harms from psychological treatments, such as ACT, are presumably low. However, the treatment objective or other related factors might induce negative feelings and expectations, highlight the fact that pain has had a major negative impact on life, and for some even provoke pain. In the present study, this might be the case for those five participants reporting clinically-significant deterioration for one or two of the variables PRS (two reports) CES-DC (two reports), FDI-P (two reports) and pain intensity (one report). This together with the wide range of values observed in most variables at all assessment points illustrates clinical challenges and a need to identify subgroups of non-responders, as well as the need to develop individually-tailored interventions and conduct careful follow-up. Our findings further support the notion that future research should address the questions of what works for whom [59,60]. As seen in our evaluation of clinically-significant changes, it is evident that some adolescents and parents benefit greatly from the interventions, whereas some do not. In a recent study of pain and functional disability trajectories during Internet-delivered CBT-treatment in an adolescent sample with chronic pain, the authors also note improvements for some participants and worsening or minimal improvements for others [61]. To date, we lack sufficient evidence for which components of treatment are related to change or which characteristics of pediatric patients suffering from chronic pain predict or moderate outcomes of both traditional CBT- and ACT-based interventions. Designing future studies to assess the details regarding change processes, including mediators and moderators of treatment outcome, is important. On a final note, it is crucial to conduct long-term follow-up. Currently, the evidence for the maintenance over time of short-term improvements in pediatric pain and disability is limited for psychological therapies overall [5], and although improvements seen post-treatment have been sustained at follow-up in previous ACT-studies [9,10,11,13,14], no study including pediatric participants with chronic pain has been conducted following up on ACT-treatment outcomes >1 year.

## 5. Conclusions

Significant improvements following an ACT-based interdisciplinary outpatient program were seen in adolescent pain interference, pain reactivity, depressive symptoms and psychological flexibility post-treatment. Furthermore, their parents reported decreased pain reactivity and increased psychological flexibility after completing a brief ACT-based parental support program. Clinically significant changes occurred for a large proportion of participants. Similar effects were seen for individual and group formats. Though tentative due to the study design, our findings provide further support for the utility of ACT to improve adolescent and parent functioning in pediatric chronic pain.

## Figures and Tables

**Table 1:** Interdisciplinary ACT-based outpatient treatment

**Prior to treatment:** Assessment of pain and pain-related disability through semi-structured screening interviews with psychologist, pain physician and physiotherapist (3 × 1 h) followed by team discussion regarding suitability for treatment; feedback to the patient and a joint decision regarding the initiation of treatment.		
**Pre-treatment assessments**		
	**Adolescent session**	**Parent session**
**Sessions 1–3: Preparing for behavior change.**	(1) Introduction to behavior analysis of difficult pain-related situations, such as ABC-analysis * with antecedent (2) Pain education with physician directed towards adolescents (e.g., information about the pain system and differences between adaptive avoidance reactions to acute pain and potentially dysfunctional avoidance reactions to long-term pain).	(3) Pain education with physician directed towards parents.
**Sessions 4–6: Shifting perspective.**	(4) Individual life values: What is important in life? How have previous strategies to avoid pain and distress led away from a valued life? (5) Introduction to the concept of increased functioning also in the presence of persisting pain.	(6) Introduction to ABC-analysis of difficult pain-related parent-child situations. Pain reduction as opposed to valued living. Clarification of parental values. Being an effective coach to your child. Home assignment: practice ABC-analyses on parent-child interactions.
**Mid-treatment assessments**		
**Sessions 7–8: Acceptance and cognitive defusion.**	(7) Evaluation of previous strategies and creative hopelessness (i.e., how have previous attempts at symptom reduction prevented a valued living). (8) Acceptance and cognitive defusion.	
**Sessions 9–17: Values-oriented behavioral activation.**	(9) Goal setting, gradual behavior activation and exposure to previously-avoided situations, in line with life values. (10) Physiotherapist: Goal setting focused on physical activities in line with chosen values. (12) Physician: Joint session. Continued pain education and discussion about symptoms in relation to behavior change. (13) Exposure, continued. (14) Physiotherapist: Evaluation and gradual increase of values oriented physical activities. (15) Exposure, continued. (16) Recruiting family and friends for support. (17) Formulating individual plan for relapse prevention and summary of treatment.	(11) Practice of acceptance and defusion in order to facilitate behaviors in line with long-term goals and values also in the presence of own worry and distress. Follow up on parent-as-coach. (12) Physician: Joint session.
**Post-treatment assessments: Concluding team session together with both adolescent and parent.**		

* ABC-analysis: (A) antecedent, (B) behavior, (C) short- and long-term consequences.

**Table 2:** Demographic and medical data for adolescents and parents.

	Total Sample	Group Condition	Individual Condition
**Children**	**30**	**12**	**18**
**Age *m* (*SD*)**	16.0 (1.6)	16.3 (1.5)	15.8 (1.6)
**Gender**			
Girls *n* (%)	24 (80.0)	11 (91.7)	13 (72.2)
Boys *n* (%)	6 (20.0)	1 (8.3)	5 (27.8)
**Pain characteristics**			
Head *n* (%)	27 (90.0)	10 (83.0)	17 (94.0)
Abdominal *n* (%)	12 (40.0)	4 (33.0)	8 (44.0)
Back *n* (%)	13 (43.0)	7 (58.0)	6 (33.0)
Joint *n* (%)	5 (17.0)	4 (33.0)	1 (5.5)
Other (e.g., parts of limbs) *n* (%)	18 (60.0)	9 (75.0)	9 (50.0)
CRPS ^a^ *n*(%)	1 (3.3)	-	1 (5.5)
Widespread *n* (%)	6 (20.0)	4 (33.0)	2 (11.0)
Pain locations > 3 *n* (%)	16 (53.0)	8 (67.0)	8 (44.0)
Pain duration in months *m* (*SD*)	57.87 (49.5)	43.18 (36.3)	67.35 (55.4)
Pain duration ≥36 months *m* (*SD*)	17 (60.7)	6 (54.5)	11 (64.7)
Current pain intensity (0–6) *m* (*SD*)	3.31 (1.4)	3.75 (1.0)	3.00 (1.7)
Continuous pain *n* (%)	22 (73.3)	10 (83.3)	12 (66.7)
Pain every day *n* (%)	5 (16.7)	2 (16.7)	3 (16.7)
Pain every week *n* (%)	3 (10.0)	-	3 (16.7)
**Current pain medication *n* (%)**	15 (50.0)	6 (50.0)	9 (50.0)
**School absence *n* (%)**			
None	4 (13.3)	3 (25.0)	1 (5.6)
Moderate	16 (53.3)	4 (33.3)	12 (66.7)
Extensive (>1 day/week)	4 (13.3)	3 (25.0)	1 (5.6)
Total absence	4 (13.3)	1 (8.3)	3 (16.7)
N/A	1 (3.3)	1 (8.3)	1 (5.6)
**Parents**	**28**	**12**	**16**
Mothers *n* (%)	24 (86.0)	10 (83.3)	14 (87.5)
Age *m* (*SD*)	47.3 (4.8)	48.42 (4.5)	46.5 (5.0)
Parent pain duration ≥1 year *n* (%)	16 (57.1)	7 (58.3)	9 (56.2)
**Marital status *n* (%)**			
Married	14 (50.0)	6 (83.0)	8 (50.0)
Co-habiting	6 (21.4)	2 (16.7)	4 (25.0)
In a relationship	2 (7.1)	1 (8.3)	1 (6.3)
Single	6 (21.4)	3 (25.0)	3 (18.8)
**Educational status *n* (%)**			
Basic/high school	16 (57.1)	5 (41.7)	11 (68.8)
University studies	12 (42.9)	7 (58.3)	5 (31.3)
**Occupational status *n* (%)**			
Full time work/study	20 (71.4)	9 (75.0)	11 (68.8)
Part time work/study	5 (17.9)	-	5 (31.3)
Not working/studying	3 (10.7)	3 (25.0)	-

^a^ CRPS = Complex Regional Pain Syndrome; *m* = mean; *SD* = standard deviation.

**Table 3:** Median (Md) and min–max scores for adolescent and parent variables at pre-, mid- and post-treatment assessment.

Outcome Variable		Pre-Md (Min–Max)	Mid-Md (Min–Max)	Post-Md (Min–Max)
**Children**				
PII (0–36)	Total	24.5 (5–35)	20.0 (4–35)	12.5 (1–35)
	Group	24.5 (11–35)	22.5 (9–35)	13.5 (6–35)
	Individual	22.5 (5–34)	18.0 (4–30)	11.0 (1–32)
PRS (0–30)	Total	21.5 (13–29)	21.0 (10–30)	13.0 (0–29)
	Group	20.0 (14–27)	21.5 (11–30)	15.0 (8–29)
	Individual	23.0 (13–29)	16.0 (10–29)	10.0 (0–28)
CES-DC (0–60)	Total	28.0 (10–47)	27.0 (15–52)	20.0 (6–47)
	Group	26.0 (10–47)	30.5 (15–52)	22.0 (9–47)
	Individual	28.5 (12–45)	26.0 (16–46)	17.0 (6–46)
FDI-P (0–60)	Total	15.5 (3–57)	10.5 (0–37)	6.0 (0–39)
	Group	19.0 (9–39)	9.5 (6–36)	6.5 (0–34)
	Individual	15.0 (3–57)	11.0 (0–37)	6.0 (0–39)
PIPS (12–84)	Total	54.0 (27–81)	49.5 (33–72)	37.0 (17–75)
	Group	54.0 (27–81)	51.0 (33–72)	40.5 (17–45)
	Individual	55.5 (38–76)	48.0 (35.50–71)	32.5 (22–65)
Pain intensity (0–6)	Total	4.0 (0–6)	4.0 (1–6)	3.0 (0–6)
	Group	4.0 (2–6)	4.0 (3–6)	4.0 (1–5)
	Individual	3.0 (0–6)	3.0 (1–6)	2.0 (0–6)
**Parents**				
HADS (0–42)	Total	15.0 (0–31)	17.0 (0–30)	13.5 (0–32)
	Group	13.5 (4–19)	12.5 (0–28)	17.0 (0–23)
	Individual	17.0 (0–31)	20.0 (6–30)	10.5 (0–32)
HADS-A (0–21)	Total	9.0 (0–17)	9.5 (0–18)	7.5 (0–16)
	Group	7.5 (4–13)	6.0 (0–17)	6.5 (0–15)
	Individual	11.0 (0–17)	10.5 (3–18)	7.5 (0–16)
HADS-D (0–21)	Total	5.5 (0–14)	8.5 (0–15)	3.5 (0–16)
	Group	4.5 (0–12)	6.0 (0–11)	7.0 (0–12)
	Individual	5.5 (0–14)	9.0 (0–15)	3.0 (0–16)
PRS-P (0–30)	Total	22.5 (5–30)	20.0 (4–30)	15.0 (1–28)
	Group	22.0 (13–30)	15.0 (5–23)	14.0 (5–28)
	Individual	22.5 (5–30)	24.0 (4–30)	15.0 (1–28)
PPFQ (0–60)	Total	32.0 (9–51)	38.0 (6–48)	42.0 (15–55)
	Group	34.5 (19–51)	40.0 (31–47)	42.0 (23–55)
	Individual	24.5 (9–46)	25.5 (6–48)	41.5 (15–54)

Measurement abbreviations: PII, Pain Interference Index; PRS, Pain Reactivity Scale; CES-DC, Center for Epidemiological Studies Depression Scale Children; FDI-P, Functional Disability Inventory Parent rating; PIPS, Psychological Inflexibility in Pain Scale; HADS, Hospital Anxiety and Depression Scale; HADS-D, depression subscale; HADS-A, anxiety subscale; PRS-P, Pain Reactivity Scale Parent; PPFQ, Parent Psychological Flexibility Questionnaire.

**Table 4:** Treatment effects for the total sample, for all time points, including *z*-scores, *p*-values, effect sizes and clinically significant changes.

Outcome Variable	Wilcoxon Signed Rank Test Pre–Mid Change	Wilcoxon Signed Rank Test Mid–Post Changes	Wilcoxon Signed Rank Test Pre–Post Changes	Effect Size (*r*) ^a^ Pre–Post	Clinically Significant Change ^b^ Pre–Post	Deterioration ^c^ Pre–Post
**Children**						
**PII**	*z* = −2.203, *p* = 0.026	*z* = −2.962, *p* = 0.002 *	*z* = −3.949, *p* < 0.001 *	*r* = −0.51	14 of 30	-
**PRS**	*z* = −0.930, *p* = 0.362	*z* = −3.651, *p* < 0.001 *	*z* = −3.765, *p* < 0.001 *	*r* = −0.49	14 of 29	2 of 29
**CES-DC**	*z* = −1.264, *p* = 0.213	*z* = −3.597, *p* < 0.001 *	*z* = −2.788, *p* = 0.004 *	*r* = −0.37	11 of 28	2 of 28
**FDI-P**	*z* = −2.584, *p* = 0.008 *	*z* = −0.142, *p* = 0.901	*z* = −2.134, *p* = 0.032	*r* = −0.35	4 of 19	2 of 19
**PIPS**	*z* = −2.199, *p* = 0.027	*z* = −4.314, *p* < 0.001 *	*z* = −4.607, *p* < 0.001 *	*r* = −0.59	19 of 30	-
**Pain intensity**	*z* = −0.525, *p* = 0.697	*z* = −1.206, *p* = 0.255	*z* = −0.980, *p* = 0.346	*r* = − 0.13	4 of 26	1 of 26
**Parents**						
**HADS**	*z* = −0.299, *p* = 0.777	*z* = −0.142, *p* = 0.899	*z* = −0.222, *p* = 0.838	-	-	-
**HADS-A**	*z* = −0.916, *p* = 0.373	*z* = −0.234, *p* = 0.836	*z* = −0.843, *p* = 0.413	-	-	-
**HADS-D**	*z* = −0.643, *p* = 0.537	*z* = −0.114, *p* = 0.933	*z* = −0.694, *p* = 0.508	-	-	-
**PRS-P**	*z* = −2.138, *p* = 0.031	*z* = −2.987, *p* = 0.001 *	*z* = −3.672, *p* < 0.001 *	*r* = −0.57	16 of 21	-
**PPFQ**	*z* = −2.315, *p* = 0.019	*z* = −3.144, *p* = 0.001 *	*z* = −4.117, *p* < 0.001 *	*r* = −0.62	12 of 22	-

^a^ for *r*, effect sizes >3 are considered of medium size and >5 large; ^b^ clinically-significant changes are defined as a change of >2 *SD* in the direction of functionality; ^c^ deterioration is defined as a change of >2 *SD* in the direction of dysfunction; * *p* < 0.01, two-tailed.
